# What Are the Prospects for Controlling Hepatitis C?

**DOI:** 10.1371/journal.pmed.1000096

**Published:** 2009-06-16

**Authors:** Paul Klenerman, Vicki Fleming, Ellie Barnes

**Affiliations:** Peter Medawar Building for Pathogen Research and National Institute for Health Research Biomedical Research Centre, University of Oxford, Oxford, United Kingdom

**Linked Research Article**

This Perspective discusses the following new study published in *PLoS Medicine*:

Drexler JF, Kupfer B, Petersen N, Grotto RMT, Rodrigues SMC, et al. (2009) A novel diagnostic target in the hepatitis C virus genome. PLoS Med 6(2): e1000031. doi:10.1371/journal.pmed.1000031

Christian Drosten and colleagues develop, validate, and make openly available a prototype hepatitis C virus assay based on the conserved 3′ X-tail element, with potential for clinical use in developing countries.

May 19 this year marked World Hepatitis Day [1].This event does not usually make the headlines in the same way that World AIDS Day does, but viral hepatitis affects about half a billion people globally (perhaps one in 12 of the global population), and so the relative publicity associated with World Hepatitis Day does not accurately reflect the importance of hepatitis as a public health problem.

The two major hepatitis viruses—hepatitis C virus (HCV) and hepatitis B virus (HBV)—share a number of features. Both viruses are readily spread through the transfer of infected blood or blood products. Both cause persistent infections and share an insidious progression after decades of asymptomatic carriage that creates a huge burden of end-stage liver disease and liver cancer. Thus, both viruses are major public health problems across the globe. However, there are substantial differences between these infections in terms of the risk groups affected, the geographical distribution of the viruses, and the tools at our disposal to deal with them.

## Prospects for Controlling HBV and HCV

For HBV we have a well-established vaccine and an emerging panel of well-tolerated oral agents for the treatment of chronic infection. Although there is still a massive burden of complex and severe infection to tackle, the pathway towards effective combination therapy has already been trodden in HIV, and careful clinical trials in this area for HBV should bring some clarity. Delivery of such drug combinations in resource-poor settings where the prevalence of carriage is high will create its own significant challenges.

For HCV we have no current vaccine, and current therapies are toxic, complex, and expensive, as well as only partially effective. Treatment is further complicated by HIV coinfection, which is increasingly encountered in some risk groups [2]. So why is the prevention and treatment of HCV infections apparently so far behind that of HBV infections? One reason is that HCV was only identified in 1989, and only successfully cultured in 2005 [3],[4]. However, the major biological hurdle to controlling HCV is the huge diversity of the virus, both within patients and among populations [5].

**Figure 1:**
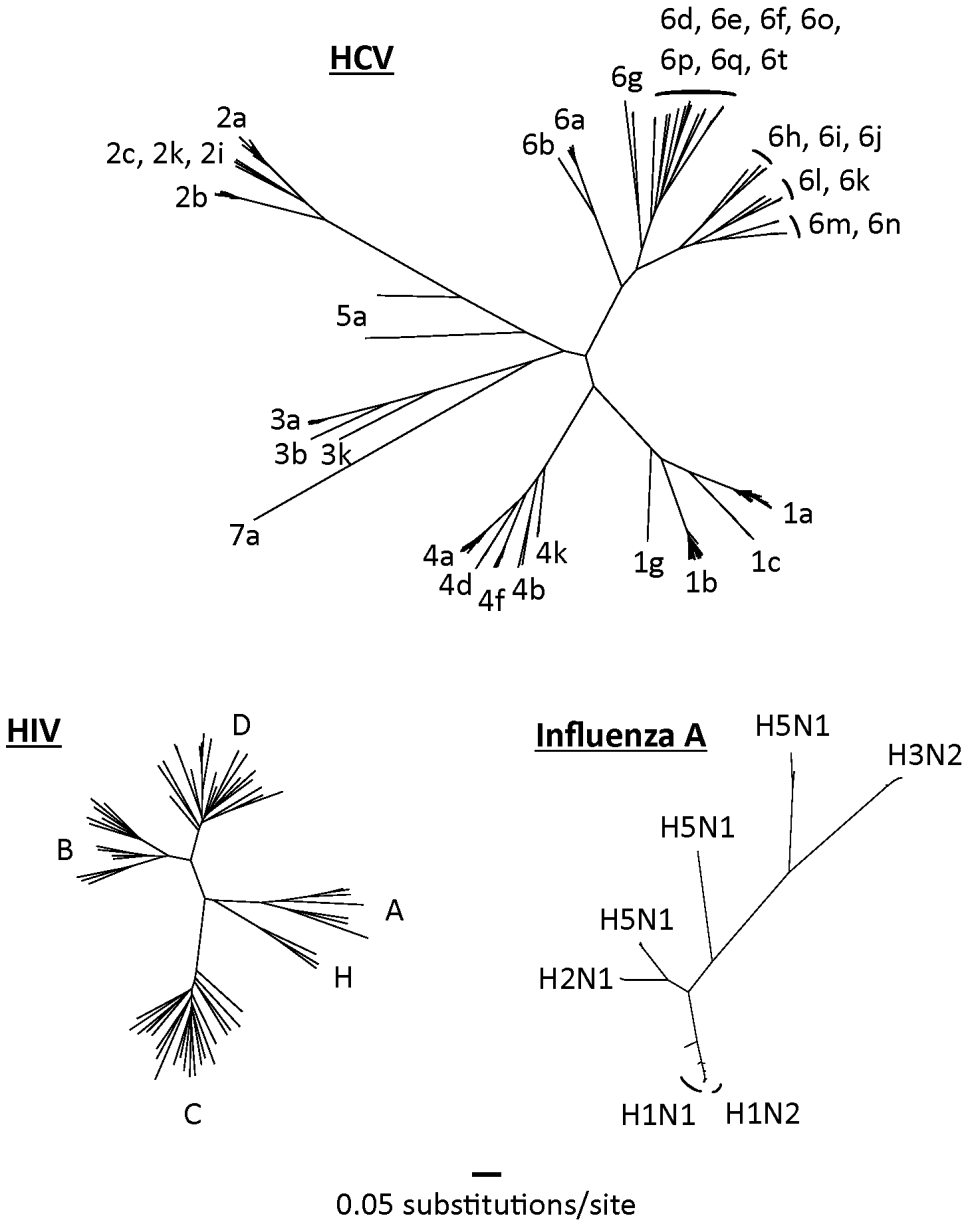
Complete genome trees of the hepatitis C virus, HIV-1 (M-group), and the hemagglutinin region of influenza A. Nucleotide sequences were randomly selected from their respective databases representing each of the major subtypes from each virus [17]–[19]. Only non-recombinant genomes were included. Maximum likelihood trees were built using GARLI (Genetic Algorithm for Rapid Likelihood Inference, available at http://www.nescent.org/). Trees have been drawn to the same scale.

HCV is an RNA-based virus with a variable genome and the capacity to evolve over time to evade drug and immunologic pressure. HCV has coevolved with human populations for centuries, if not millennia [6], and has diversified widely over this period (Figure 1). By comparison, the phylogenetic tree of HIV is much more compact because this virus has had less than a century in which to diversify in humans.

The net result of this diversification is the existence of seven major genotypes of HCV (the last added very recently) that share less than 80% sequence homology with one another, and more than 50 HCV subtypes [7]. Although these genotypes may have arisen over long periods as endemic strains in geographically distinct regions (e.g., genotype 6 in southeast Asia [8]), most have now spread globally. Genotype 1 is particularly common in western Europe and the United States, although genotype 3 is also now very common in the United Kingdom as a result of its spread through intravenous drug-using populations and through immigration from the Indian subcontinent.

Multiple genotypes occur in many other viruses, including HBV, but their importance in HCV is particularly high because both the duration and success rate of current treatments for HCV infection (pegylated interferon-alpha and ribavirin) are highly genotype dependent. Thus, genotypes 2 and 3 are typically associated with much greater response rates than genotypes 1 and 4 (70%–80% long-term clearance versus 40%–50%) and require shorter treatment periods (six months versus one year) [9]. The biological basis for these differences is unclear—the genomes of these genotypes are so diverse that such differences could result from multiple complex changes. Even within a single genotype (e.g., genotype 1), the fundamental mechanisms behind relative resistance to treatment of different HCV subtypes are not fully defined, although an interferon-sensitivity determining region has been described [10].

## The Role of Nucleic Acid Tests for HCV

Given these important clinical and virologic differences between HCV genotypes, robust and sensitive nucleic acid tests for HCV have a major role to play in virus detection and in guiding treatment and thus are at the core of current clinical practice in developed countries. However, these tests are relatively complex molecular tests and are therefore not universally available. Additionally, they may not be equally sensitive at detection of all genotypes. In a recent article in *PLoS Medicine*, however, Christian Drosten and colleagues described a new approach to nucleic acid testing in HCV [11].

The authors generated a test based on a highly conserved region in the 3′ end of the virus (most current tests are based on the 5′ end) and validated their assay to show that it was sensitive in detection of a wide range of genotypes from geographically diverse populations. They also attempted to reduce the overall cost of their approach and have thus provided a novel system that uses an open (i.e., non-proprietary) protocol that might be particularly appropriate for resource-poor settings. This new assay is potentially an important step forward for laboratories in such regions and, if rolled out effectively, could provide novel information relevant to the prevalence, clinical impact, and treatment response of HCV genotypes that are currently poorly studied—most clinical analyses, and vaccine and treatment trials have focused on genotype 1.

Although very simple and cost-effective tests to detect, quantify, or genotype HCV in resource-poor areas could be of great value in future, the overall costs and usefulness of any such test in comparison to other methods and in relation to other public health priorities in such regions will need to be considered carefully. Thus, although conventional PCR methods as used by Drosten and colleagues look promising, non-PCR-based methods such as loop-mediated isothermal amplification (LAMP) also need to be considered, since little specialist equipment is required for LAMP and the sensitivity appears to be high [12]. In the end, however, the definitive test for any new method of HCV analysis will be clinical utility in the field.

## The Extreme Viral Diversity of HCV

As we mark World Hepatitis Day, the recent paper by Drosten and colleagues once more draws our attention to one of the key features of HCV: its extreme viral diversity, which brings enormous challenges for the future. The capacity for HCV to evolve creates a complex target for both vaccine and drug development. Nevertheless, recent advances in both these areas provide some cautious hope for the future—at least in the case of genotype 1 infection [13],[14]. Key to successful vaccine development will be the generation of effective, sustained, and broad anti-HCV immune responses. However, the immune responses to non-genotype 1 viruses are very poorly described, and recent data suggest that there is relatively little overlap between immune responses to genotypes 1 and 3. Thus, at present it is unclear whether HCV vaccines against specific genotypes will provide any cross-protection against other genotypes [15]. The situation with drugs may be even more complex, with pre-existing diversity even within genotype 1 already providing some level of drug resistance [16].

Future studies of the diverse HCV genotypes that exist globally—hopefully facilitated by the recently published methods—will, therefore, help us understand the overall clinical impact of HCV in affected populations and will determine our potential to intervene. Since HCV emerged from the shadows 20 years ago, it has shown itself to be “smarter than the average virus.” Thus, it may take longer than 20 years for us to put it back into the shadows, and it will probably take all our efforts to do so.

## Abbreviations

HBV: hepatitis B virus
HCV: hepatitis C virus

## References

[1] World Hepatitis Alliance (2009). World Hepatitis Day.. http://www.worldhepatitisday.org/.

[2] Klenerman P, Kim A (2007). HCV–HIV coinfection: Simple messages from a complex disease.. PLoS Med.

[3] Houghton M (2009). Discovery of the hepatitis C virus.. Liver Int.

[4] Wakita T, Pietschmann T, Kato T, Date T, Miyamoto M (2005). Production of infectious hepatitis C virus in tissue culture from a cloned viral genome.. Nat Med.

[5] Simmonds P (2004). Genetic diversity and evolution of hepatitis C virus—15 years on.. J Gen Virol.

[6] Pybus OG, Charleston MA, Gupta S, Rambaut A, Holmes EC (2001). The epidemic behavior of the hepatitis C virus.. Science.

[7] Kuiken C, Simmonds P (2009). Nomenclature and numbering of the hepatitis C virus.. Methods Mol Biol.

[8] Pybus OG, Barnes E, Taggart R, Lemey P, Markov PV (2009). Genetic history of hepatitis C virus in East Asia.. J Virol.

[9] Zeuzem S, Berg T, Moeller B, Hinrichsen H, Mauss S (2009). Expert opinion on the treatment of patients with chronic hepatitis C.. J Viral Hepat.

[10] Torres-Puente M, Cuevas JM, Jimenez-Hernandez N, Bracho MA, Garcia-Robles I (2008). Genetic variability in hepatitis C virus and its role in antiviral treatment response.. J Viral Hepat.

[11] Drexler JF, Kupfer B, Petersen N, Grotto RMT, Rodrigues SMC (2009). A novel diagnostic target in the hepatitis C virus genome.. PLoS Med.

[12] Nagamine K, Hase T, Notomi T (2002). Accelerated reaction by loop-mediated isothermal amplification using loop primers.. Mol Cell Probes.

[13] Thompson AJ, McHutchison JG (2009). Review article: Investigational agents for chronic hepatitis C.. Aliment Pharmacol Ther.

[14] Thimme R, Neumann-Haefelin C, Boettler T, Blum HE (2008). Adaptive immune responses to hepatitis C virus: From viral immunobiology to a vaccine.. Biol Chem.

[15] Schulze Zur Wiesch J, Lauer GM, Timm J, Kuntzen T, Neukamm M (2007). Immunologic evidence for lack of heterologous protection following resolution of HCV in patients with non-genotype 1 infection.. Blood.

[16] Gaudieri S, Rauch A, Pfafferott K, Barnes E, Cheng W (2009). Hepatitis C virus drug resistance and immune-driven adaptations: Relevance to new antiviral therapy.. Hepatology.

[17] Bao Y, Bolotov P, Dernovoy D, Kiryutin B, Zaslavsky L (2008). The influenza virus resource at the National Center for Biotechnology Information.. J Virol.

[18] Combet C, Penin F, Geourjon C, Deleage G (2004). HCVDB: Hepatitis C virus sequences database.. Appl Bioinformatics.

[19] Division of AIDS, National Institute of Allergy and Infectious Diseases (2009). HIV databases.. http://www.hiv.lanl.gov/.

